# Pierre Curie, 1859–1906

**DOI:** 10.3747/co.2007.110

**Published:** 2007-04

**Authors:** R.F. Mould

**Keywords:** Pierre Curie, piezoelectricity, radium, radioactivity

## Abstract

The year 2006 marked 100 years since the death of Pierre Curie. It is therefore appropriate that we remember his life and his work, which was cut short by his untimely death from an accident on the Pont Neuf, Paris, on April 19, 1906. He had already accomplished much during his life, both before the discovery of radium with Marie Curie, in work co-authored with his brother Jacques on piezoelectricity, and afterwards, when he published the results of several experimental studies with radium and radon. He came from a medical family, and his grandfather Pierre Curie was a famous homeopathic physician. He has, in print, unfairly been relegated to the background—his own scientific contributions having been overtaken by the fame of Marie Curie, probably because she outlived him by 28 years.

## 1. INTRODUCTION

Only a single biography 1 has been written on Pierre Curie, as compared with more than ten on Marie Curie. Pierre Curie’s papers were published in 1908 2, after his death, because such publication was then the policy in Paris for many famous scientists.

Today, it is largely forgotten that the Curie brothers discovered piezoelectricity 3–5, a phenomenon that enabled the development of the piezoelectrometer. That instrument was used for the early scientific measurements of radium preparations. Pierre Curie was instrumental with Marie Curie in discovering both polonium 6 and radium 7, but besides his several joint papers with her, he also published alone and with other authors. The division of work between the Curies was largely split into chemistry for Marie and physics for Pierre.

## 2. THE CURIE FAMILY

The Curie family originated from Mulhouse in Alsace before the German annexation of Alsace and Lorraine at the end of the nineteenth century. Pierre Curie’s grandfather, Paul Étienne François Gustave, and father, Eugène, were physicians. Eugène was brought up in Paris and studied natural sciences and medicine at the Musée d’Histoire naturelle at 47 rue Cuvier where, later, Henri Becquerel was to be professor of physics and to discover radioactivity. It was also in a house in the rue Cuvier, number 16, that Pierre Curie was born. Later, at 12 rue Cuvier, the Laboratoire Curie was established by the Sorbonne in 1904. The laboratory remained on that site until 1914, when the Institut du Radium was built in rue Pierre Curie.

**FIGURE 1:**
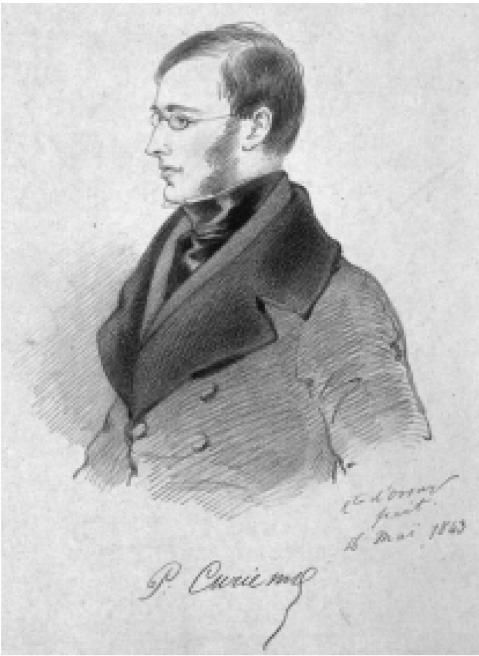
Paul Curie from a lithograph by C. Graf in 1843.

**FIGURE 2:**
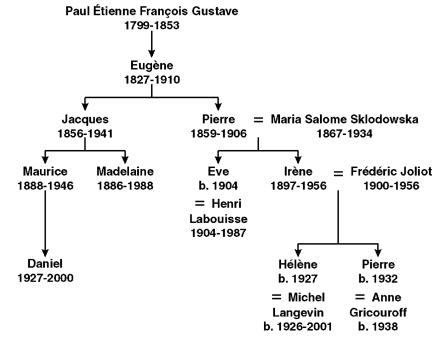
The Curie family tree.

Of the first three generations in the family, only Eugène Curie never published. Paul established himself as a physician in England working at the London Homeopathic Infirmary 8–12 having previously worked as a surgeon in the Military Hospital of Paris (Figure 1). In the Curie family tree shown in Figure 2, only Eve Curie was not a scientist; however, she was the author of the first biography of Marie Curie 13. Maurice and Daniel were both physicists. Hélène is a nuclear physicist, and Pierre, a biochemist.

In 1903, Pierre and Marie, with Henri Becquerel, were awarded the Nobel Prize in Physics. In 1911, Marie was awarded the Nobel Prize in Chemistry. In 1935, Irène and Frédéric were awarded the Nobel Prize in Chemistry. In 1965, Henri Labouisse received the Nobel Peace Prize on behalf of unicef.

Starting in 1913, Maurice Curie 14–17 worked for a year with Marie Curie in the Laboratoire Curie in the rue Cuvier, and throughout World War I, he corresponded with her. He spent 12 months on the front line before 1917, mostly near Verdun, but he survived the war. Son Daniel published with his father 17,18.

Pierre’s death was reported on April 20, 1906, in the French newspaper *Le Matin.* The headline read “M. Pierre CURIE, le savant qui découvrit le radium, a été écrasé dans la rue et tué net par un camion.... M. Curie traversait la rue Dauphine, près du Pont-Neuf, à deux heures et demie, se tenant derrière un flacre. A ce moment arrivait du Pont-Neuf un camion attelé de deux chevaux et chargé d’équipements militaires, conduit par le charretier Louis Manin ...” 19 Rain was heavy at the time, and it was assumed that Pierre’s sight was obscured by his umbrella and that he saw too late the heavy wagon approaching him.

## 3. ALFRED CURIE

**FIGURE 3:**
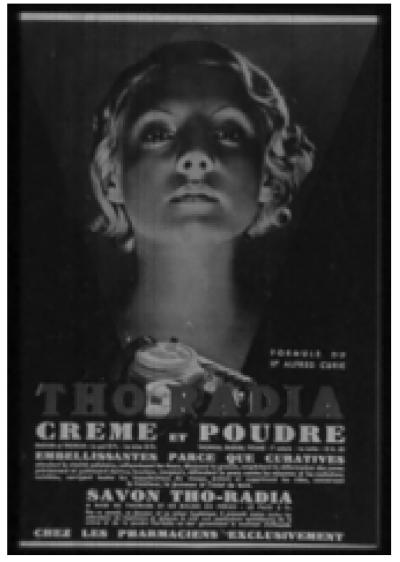
Advertisement for Tho-Radia face cream and powder.

It should perhaps also be noted that the Dr. Alfred Curie who is said to be the inventor of the formula for the cosmetic Tho-Radia face cream (Figure 3) was not a member of Pierre Curie’s family—although he was indeed a Curie.

For many years, even the director of the Musée Curie in Paris assumed that he was “un médecin qui n’a jamais existé!” 20 And all radium historians until 2003 also considered that he was a mythical person used only for the promotion of beauty products in the 1920s (Huchette N, Musée Curie. Personal communication, October 2003). However, it now transpires that, although the mysterious Alfred was not related to Pierre Curie, he nevertheless existed 21, and in 1911, for his doctorate in medicine from the University of Paris, he submitted a thesis titled *Treatment of Spina Bifida* 22. He was traced through annual copies of *Le Guide Rosenwald,* which lists all French doctors and which shows that, in 1912, he was an internist at L’Hôpital de Clichy. Afterwards, he seems to have been in general practice, having set up a private clinic. He was born in 1973 and died in Paris in 1940 21.

Whether it was really Alfred Curie—or an Egyptian pharmacist, Alexis Moussalli—who invented the formula is not known, but nevertheless a formula for Tho-Radia did actually exist 21. For the face cream, the radioactivity consisted of 0.5 g thorium chloride and 0.25 mg radium bromide per 100 g of cream. The Tho-Radia patent (“marque”) was registered by the Tribunal de Commerce de la Seine on November 29, 1932.

It is probable that the commercial mastermind behind Tho-Radia was Alexis Moussalli. This doctor of pharmacy, who had been born in Cairo, Egypt, in 1894, submitted his thesis to the University of Nancy on the subject of “le bacille pyocyanique et les bacilles de la dysenterie” 23. During the period 1927–1934, he patented 101 preparations based on radium or radioactive substances, but only Tho-Radia and Crème Radio-Thorium used a formula devised by Alfred Curie. The other preparations included Laboradium, Microradium, Radiobust, Radiofluide, Radioskin, Radium Cure, and Radiviril. Moussalli ceased his activities in 1956, although it is unknown if that was the year he died.

## 4. PIEZOELECTRICITY AND CRYSTALLOGRAPHY

Pierre received his early education at home before entering the Faculty of Sciences at the Sorbonne and obtaining his Licence in Physics in 1878. He then continued as a demonstrator in the Sorbonne’s physics laboratory, where he worked with his brother Jacques. In 1883, Jacques left to take up the head lectureship in mineralogy at the University of Montpellier. Montpellier being in the south of France and very distant from Paris, Jacques’ move ended the scientific collaboration of the brothers, but not before they had completed important studies on the electric properties of crystals and had discovered piezoelectricity 3–5.

In 1883, Pierre also left the Sorbonne. He took charge of all the practical work (Chef de travaux pratique) and eventually became Professeur de Physique Générale et Electricité Théorie at the École Supérieure de Physique et de Chimie Industrielles (espci), which until 1946 was called the École Municipale de Physique et de Chimie Industrielles. He was to remain there for 22 years, obtaining his Doctor of Science degree in 1895 24. In 1900, he returned to the Sorbonne as a professor in the Faculty of Sciences.

As early as the eighteenth century, the crystals of some minerals were known to generate an electric charge when heated; the phenomenon was called pyroelectricity. The phenomenon of piezoelectricity (named after the Greek *piezein,* meaning to squeeze or press) was discovered by the Curie brothers when they showed that crystals of tourmaline, quartz, topaz, cane sugar, and Rochelle salt generated electrical polarization under mechanical stress. Piezoelectricity from a thin slice of quartz was the basis for the Curie electrometer.

The first practical application of the discovery (after the measurement of radioactivity) was in a sonar piezoelectric device. This ultrasonic submarine detector was developed in 1917—during World War I—by Paul Langevin, who had been one of Pierre’s students at espci. The device consisted of a transducer (made of thin quartz crystals carefully glued between two steel plates) and a hydrophone to detect the returning echo. The transducer emits a high-frequency note of sound. By measuring the time taken to hear the echo of the sound waves returning from an object, the distance to the object can be calculated.

## 5. THE CURIE POINT

Pierre Curie’s name is not only commemorated by the Curie piezoelectrometer, but also by the Curie point (or Curie temperature) of ferromagnetic material. The Curie point is the temperature above which the material loses its characteristic ferromagnetic properties. In an analogy to ferromagnetism, the Curie point is also used to describe the temperature above which piezoelectric materials lose their spontaneous polarization and piezoelectric properties. During the period 1880–1895, without any collaboration with his brother, Pierre Curie undertook further research into piezoelectricity, crystal symmetry, and the Curie point 25–28.

## 6. POLONIUM AND RADIUM

In 1898, Pierre Curie ceased his studies on piezoelectricity and crystallography to be able to concentrate solely on working with Marie Curie—a collaboration that culminated in the discovery of polonium and radium 6,7. This phase of Pierre’s life is very well documented not only in scientific publications, but also in popular magazines and journals. Invariably, the latter were illustrated with not-very-good representations of Pierre and Marie working together in their rue Lhomond laboratory.

In his Nobel address, Pierre Curie made the following famous statement:

It is possible to conceive that, in criminal hands, radium might prove very dangerous, and the question therefore arises whether it be to the advantage of humanity to know the secrets of nature, whether we will be sufficiently mature to profit by them, or whether that knowledge may not prove harmful.

Take for instance the discoveries of Nobel: Powerful explosives have made it possible to achieve admirable things, but they are also a terrible means of destruction in the hands of those great criminals who draw nations into war. I am among those who believe with Nobel that humanity will obtain more good than evil from future discoveries (Curie P. Radioactive substances, especially radium. Presented in Stockholm, June 6, 1905).

## 7. SELF-EXPOSURE RADIUM BURNS

### 7.1 Friedrich Walkoff and Friedrich Giesel

The first self-exposure experiments were made not by Henri Becquerel, but by two Germans: Friedrich Walkoff and Friedrich Giesel. (Giesel worked for the Buchler company in Brunswick, Buchler being the first producer of radium sources in Germany.) In October 1900, Giesel described in a scientific journal the strapping of 270 mg of radium salt to his inner forearm for a duration of two hours 29; he also wrote about it to Pierre Curie (Joliot–Langevin H. Personal communication, October 2004). The Walkoff reference of 1900 30 consisted of just three lines in a three-page general review (dealing mainly with photographic questions) presented to a photographic club in Munich. The review was published in the October 1900 issue of *Photographische Rundschau: Zeitschrift für Freunde der Photographie.* The title, translated into English, is “Invisible, photographically effective rays.” I am most grateful to Dr. Jean Dutreix, for the translation and comments that follow.

Walkoff wrote:

Furthermore, radium owns astonishing physiologic properties. An exposure of the arm to two 20-minute sessions has produced an inflammation of the skin which has now lasted already for two weeks, and exhibits the same aspect as that obtained after a long exposure to X-rays. Trials still not completed, made in the Munich Hygiene Institute, seem to show an effect on micro-organisms.

An interesting question is posed (and answered below) by Dutreix: “Is this a personal experiment, or is he reporting on an experiment made by his friend Friedrich Giesel?” The details are scarce but precise: two exposures of 20 minutes and a skin inflammation now lasting for two weeks. A few lines above his radium comments, Walkoff reports on two of his own photographic experiments. Why not on the skin experiment, which is so casually presented? The first impression, therefore, is that Walkoff is mentioning an experiment undertaken by someone else.

However, the following three points incline us to believe that the two skin trials by Walkoff and Giesel are not connected and that they are two separate experiments:

- Walkoff writes: “Dr. Giesel, out of friendliness, put at my disposal 0.2 g of radium much coveted by the physicists [*physiker—*physicist, and not physician] for physiologic trials.”
- In the description of the reaction “now last[ing] already for two weeks” suggests that Walkoff is acquainted with the follow-up. Is this personal information or information from Giesel?
- Walkoff pays credit to his “friend Giesel for producing polonium and radium,” but does not mention any physiologic trials undertaken by Giesel. Because Walkoff warmly expresses his gratitude to Giesel, it can be assumed that if he were referring to an experiment by Giesel, he would have had the courtesy to mention this fact.

In conclusion, an interesting observation relating to Friedrich Giesel was later published in 1905 31 and clearly shows the hazards of commercially preparing the early radium sources: “Giesel’s breath was so radioactive that it would still discharge an electroscope 18 hours after he left his laboratory, and Giesel’s body was the most radioactive that had yet been measured.”

### 7.2 Henri Becquerel and Pierre Curie

Becquerel’s radium burn was often reported to have been “accidental” after he had placed a radium source inside his jacket pocket. However, Becquerel gave credit for the first observations to Walkoff and Giesel 29,30, and together with Pierre Curie, to whom Giesel had previously written (Joliot–Langevin H. Personal communication, October 2004), Becquerel reported his own self-exposure measurements with radium 32 with a source placed many times in a pocket of his jacket.

Marie Curie, in her biography of Pierre Curie 1, describes Becquerel’s reaction to the experience of receiving a radium burn:

In order to test the results that had just been announced by F. Giesel, Pierre Curie voluntarily exposed his arm to the action of radium during several hours. This resulted in a lesion resembling a burn that developed progressively and required several months to heal. Henri Becquerel had by accident a similar burn as a result of carrying in his vest pocket a glass tube containing radium salt. He came to tell us of this evil effect of radium, exclaiming in a manner at once delighted and annoyed: “I love it, but I owe it a grudge.”

What now follows is the English translation—for which I am most grateful to Dr. Bernard Asselain—of the text of the Becquerel and Curie paper 32, in which, toward the end, a mention is made that Marie Curie also conducted radium self-exposure experiments:

Radium radiations act energetically on the skin: the effect produced is close to that produced with Röntgen X-rays. First observations of this action are due to Walkoff and Giesel in 1900. Mr. Giesel applied to his arm for two hours, radiferous radium bromide placed in a celluloid sheet. Radiations acting through the celluloid provoked a light red colour of the skin. Two or three weeks later, the red colour increased, and an inflammation of the tissues appeared, and the skin sloughed off.

Mr. Curie reproduced on himself the Giesel experiment letting it act for 10 hours on his arm, through a thin sheet of gutta percha, the radiferous barium chloride [“Radiferous barium” is not a misprint for “radiferous radium”; the discovery of radium was associated with the barium extract from pitchblende—hence the term radiferous barium. The discovery of polonium was associated with the bismuth extract from pitchblende] of relatively low activity [approximately 5000 times the activity of metallic uranium]. After the action of the radiations, the skin became red over a 6-cm^2^ surface. The result was similar to that of a skin burn, but the skin was not painful. After some days, the red colour increased without spreading. On the 20th day, scabs were formed and then an open wound needing covering up. On the 42nd day, the epidermis started to recover around the wound, reaching the centre, and 52 days after the action of the radiations, an area of 1 cm^2^ of a grey colouration remained, indicating a deeper necrosis.

Mr. Becquerel, bringing a small sealed tube of a few decigrams of radiferous barium chloride of a high activity level [800,000 times the activity of uranium] underwent the same experiment. The radiferous product was enclosed in a sealed glass tube, and the cylindrical volume was about 10–15 mm in length and 3 mm in diameter. The tube, enclosed in a sheet of paper was inside a small cardboard box. On April 3 and 4, this small box was placed many times in a pocket of his jacket, the total time being assessed as a 6-hour exposure. On April 13, he discovered that the radiations through the tube, the box, and the clothes produced a red spot on the skin which became darker in the next days: marking in red the oblong size of the tube, with an oval shape of 6×4 cm^2^. On April 24, the skin sloughed off and then the central part became ulcerated with a discharge. The wound was treated during 1 month with a bandage with oil and calcar. Necrosed tissues sloughed off, and on May 22—that is, 49 days after the irradiation—the wound was repaired, leaving a scar marking the position of the tube.

During the treatment of the burn, a second oblong spot appeared on about May 15 at the place of the opposite corner of the jacket pocket where the radioactive tube was placed. The action was dated either at the same date as the first one, or more likely on April 11, even if it lasted a very short time, no more than one hour. Erythema thus appeared 34 days at least after the initial action. Inflammation progressed with the aspect of a superficial burn. On May 26, the skin began to slough off. With the same care as for the first one, this burn was cured more quickly. During the period of observation, on April 10, 11, and 12, the same radioactive tube was put in another pocket of the jacket, but enclosed in a lead tube of about 5 mm thickness. The tube was kept in the pocket for 40 hours, and no effect was observed.

We can add that Madame Curie, carrying in a small sealed tube only a few centigrams of the same very active substance that gave the above mentioned lesions, presented with similar burns, although this little tube was enclosed in a thin metallic box. For instance, a short action, during less than 30 minutes, produced 15 days later a red spot which was transformed into a blister similar to that due to a superficial burn that takes 15 days to be cured. These facts show that the duration of the lesion’s evolution varies according to the intensity of the radiation and the duration of the excitation action.

After these effects we have described, we experimented on our hands the different actions during researches with these very active products. Hands have a general trend to become scaly, and the extremities of the fingers which held tubes or capsules containing very radioactive products become hard and sometimes very painful. For one of us, the inflammation of the extremities of the fingers lasted about 15 days and finished when the skin dropped off, but the painful sensation did not disappear for two months.” 32

**FIGURE 4:**
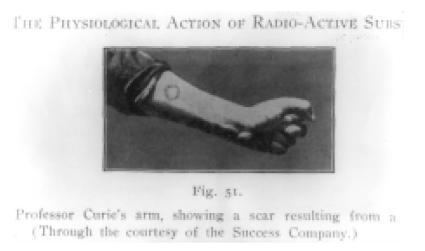
Pierre Curie’s self-exposure radium burn. It was described 33 as “Professor Curie’s arm, showing a scar resulting from a radium sore.”

Figure 4 was for many years assumed to be a newspaper photograph of Pierre Curie’s self-exposure radium burn on his forearm. That assumption was made because the Marie Sklodowska Curie Museum in Warsaw only ever showed the photograph on its own without any wording. *Comptes rendus de l’Academie des sciences* 32 gave a full description of the experiment, but did not include this photograph.

The mystery of the provenance of Figure 4 deepened when the photograph could not be located in any early textbooks on radium and X-rays held in the British Library, the British Institute of Radiology library, the library of the Welcome Institute for the History of Medicine, the Royal Society of Medicine library, or the library of the Science Museum, Imperial College, London. The provenance was solved only in 2006 when the elusive textbook 33 containing the photograph of Pierre Curie’s radium burn was found to be titled Radium and Radio-Active Substances, written in 1905 by Charles Baskerville. However, that discovery does not solve the mystery of what the Success Company was.

Baskerville commences his radium biology chapter 33 with reference to the experiences of Walkoff 29 and Giesel 30, and when referring to Figure 4, quotes “Madame Curie’s thesis” as the source for the statement “the action of radium upon the skin can take place across metal screens, but with weakened effect” 34. Indeed, Baskerville’s exact wording really is from Marie Curie’s thesis and can be found in the latest English translation (of 2002) 35, which is an unabridged republication of the English 1904 translation published by Van Nostrand in New York 36.

## 8. RESEARCH PAPERS

During the period 1900–1906, Pierre Curie published twenty-one papers (Table i), either alone or with coauthors other than Marie Curie. Only one of these was with Henri Becquerel 32 (in 1901), and that paper includes the self-exposure experiments described earlier. Those experiments in turn led Pierre Curie to loan some radium to Henri Danlos, a dermatologist at the Hôpital St-Louis, Paris, for the first radium treatment in France of (non-malignant) skin disease 37. A second paper on the physiologic action of radon (radium emanation) was published in 1904 38, demonstrating Pierre Curie’s continued interest in radium and radon therapy.

The first two topics in the list from Table i attracted much early interest. In regard to the first paper, two types of Becquerel rays, deviable and non-deviable, were initially considered to exist. The former were beta rays, and the latter, alpha rays. However, the alpha rays were non-deviable only because the tested magnetic field was not of sufficient strength for any deviation to be detected. The second paper referred to reports that radium could induce radioactivity in other materials. However, as Ernest Rutherford later demonstrated, that phenomenon, which was common to thorium and thoron and to radium and radon, was attributable to an active deposit.

In comparison with Table i, Table ii lists the topics on which Marie Curie published during 1900–1906. Of a total of thirteen papers, she is the sole author of all of them. This period also included the publication of her doctoral thesis 35,36.

**FIGURE 5:**
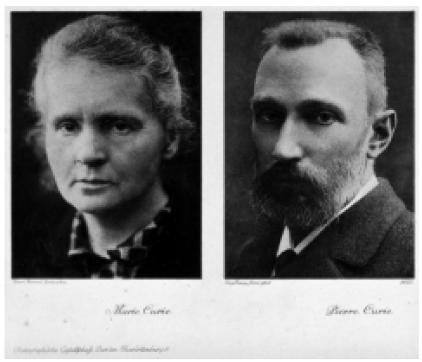
Pierre and Marie Curie.

Pierre Curie’s final paper 39 was published on June 25, 1906—some two months after his death. Figure 5 shows Marie and Pierre Curie circa 1906.

## 9. LABORATORY WORKING CONDITIONS IN 1903

In 1903, the American journalist Cleveland Moffett interviewed 40 Pierre Curie in the rue Lhomond, in the laboratory where polonium and radium had been discovered in 1898 6,7. This interview was one of the very few recorded with Pierre Curie, and Moffett’s description of an accident with radium is very interesting:

I found him in one of the rambling sheds of the École de Physique bending over a small porcelain dish, where a colourless liquid was simmering, perhaps half a teacupful, and he was watching it with concern, always fearful of some accident. He had lost nearly a decigram (1.5 grains troy) of radium only a few weeks previously, he said, in a curious way. He had placed some radium salts in a small tube, and this inside another tube, in which he created a vacuum. Then he began to heat both tubes over an electric furnace, when suddenly at about 2000°F, there came an explosion which shattered the tubes and scattered their precious contents. There was absolutely no explanation of this explosion; it was one of the tricks that radium is apt to play on you. Here his face lightened with quite a boyish smile.

One demonstration by Pierre Curie for Moffett involved the use of radium to illuminate a page for reading purposes. Moffett recorded it as follows:

M. Curie took me into a darkened room where I saw quite plainly the light from the radium tube, a clear glow sufficient to read by if the tube were held near a printed page. And of course this was a very small quantity of radium, about 6 centigram. “We estimate,” said Pierre Curie, “that a decigram of radium will illuminate 15 square inches of surface sufficient for reader.” In addition he estimated that “a kilogram [2.2 pounds] of radium would illuminate a room 30 feet square with a mild radiance; and the light would be much brighter if screens of sulphide of zinc were placed near the radium, for these are thrown by the metal into brilliant phosphorescence.” 40

Pierre Curie was well aware of the dangers involved and concluded, “For some time to come the radium light will only be a laboratory wonder.” He also gave an “outside estimate” of the amount of radium then in the world being only 4 g—one third of an ounce in France, one gram in Germany, less than one gram in the United States, and half a gram in the rest of the world—and that “you could heap it all in a tablespoon,” and “a kilogram of radium would cost about 10 million francs, and that radium is worth about 3000 times its weight in pure gold.” (Table iii gives the costs of radium for the period 1899–1938 41–47.)

Pierre Curie also mentioned 40 that he had just returned from London, lecturing at The Royal Institution, and that “his hands had become much peeled, and very sore from too much contact with radium, and for several days he had been unable to dress himself.” Earlier Moffett had also remarked on the biologic effect of radium, reporting that “Pierre Curie pulled up his sleeve and showed me a forearm scarred and reddened from fresh healed sores.”

## 10. TEACHING EXPERIMENT

**FIGURE 6:**
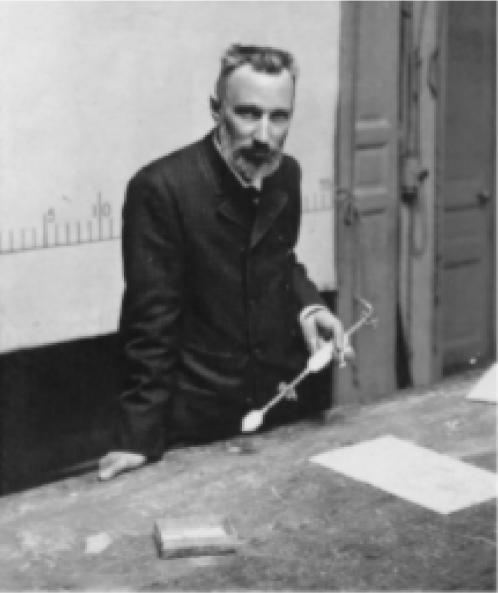
Pierre Curie teaching in 1904 at the Curie Laboratory, 12 rue Cuvier.

Table i shows the topics of Pierre Curie’s research experiments, but it should be emphasized that his professional life also included a significant amount of teaching. Figure 6 shows him in 1904 at the lecture theatre in the rue Cuvier with glassware from one of his standard experiments. He used this experiment to demonstrate for his students the phenomenon of radioactivity.

Moffett provides the only full description of this experiment 40, considering that it was “the most striking experiment” performed by Pierre Curie and the “one devised by him to prove the existence of radium emanations, a kind of gaseous product (quite different from the rays) which this extraordinary metal seems to throw off constantly as it throws off heat and light.”

**FIGURE 7:**
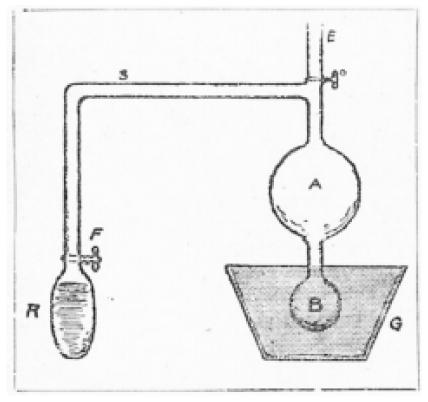
Schematic diagram of the experiment Pierre is performing in Figure 6 40.

Figure 7 is Moffett’s schematic diagram of the experiment. The experiment itself was described in these terms:

M. Curie constructed an apparatus in which a glass tube R, containing a solution of radium is connected to two glass bulbs, A and B, containing sulphide of zinc. The experiment is begun by exhausting the air from the two bulbs A and B, by means of air pump connections through the tube E. The air is not exhausted, however, from the tube R, over which the stop-cock F is closed, and within which the emanations have been allowed to accumulate. The room is now darkened, and it is seen that so long as the stop-cock F remains closed there is no glow in bulbs A and B, but as soon as the stop-cock F is opened both bulbs shine brilliantly, so that the light is plainly visible at a distance of several hundred yards. Now, obviously if this effect were due to the radium rays, it would be produced whether the stop-cock F were open or closed, since the radium rays pass freely through glass and need not follow the tube S in order to reach the bulbs A and B.

It is therefore clear that the sudden light in the bulbs is due to the passage of *something* out of the tube R, and through the tube S, that *something* being kept back by the glass of the bulb R until the stop-cock F is opened. So that we conclude that the emanations of radium *cannot* pass through glass and are a manifestation quite distinct from the rays of radium, which *can* pass through but do not influence the sulphide of zinc.

This point having been established, M. Curie proceeded to the most sensational part of his demonstration, by closing the stopcock F and then placing the lower bulb B, still radiant, in a vessel G containing liquid air, the result being that the light in the bulb B gradually grew stronger while the light in the bulb A diminished, until presently, *all* the light seemed concentrated in B and gone from A, the conclusion being that the intense cold of liquid air had produced some change in the emanations, has possibly reduced them from a gas to a liquid, this withdrawing them from A to B and checking the one glow while increasing the other. 40

Moffett’s legend to Figure 7 was “M. Curie explaining the wonders of radium at the Sorbonne. This experiment with the radium light is described in this article.”

## Figures and Tables

**TABLE I:** Topics of Pierre Curie’s research papers, 1900–1906

Action of a magnetic field on Becquerel rays
Induced radioactivity and radon
Secondary rays produced by X-rays
Physiological action of radium rays
Conductivity of liquid dielectrics
Decay of radon
Heat released by radium salts
Radioactivity of gases emitted from thermal water sources

**TABLE II:** Topics of Marie Curie’s papers, 1900–1906

Penetration of Becquerel rays undeflected by a magnetic field
Atomic weight of radium
Reviews of work on radioactive substances
Decay of polonium

**TABLE III:** Costs of 1 g radium, 1899–1938 41–47—by comparison, the Hope Diamond was sold in 1909 for US$80,060, the equivalent of US$8,779/g

*Year*	*Cost*	*Comments*
1899	£1000 rising to £12,000	Buchler Company costs quoted by Friedrich Giesel
1902	US$3,000	
1904	US$18,600	
1906	US$50,000 in April	
	US$70,000 in August	
	US$90,000 in December	
1914	US$150,000	
1921	US$100,000	This was the year that Marie Curie was given 1 g radium during her visit to the United States
1933	US$70,000	
1938	US$25,000–US$30,000	Agreement by a Belgian–Canadian cartel
